# Maitotoxin Is a Potential Selective Activator of the Endogenous Transient Receptor Potential Canonical Type 1 Channel in *Xenopus laevis* Oocytes

**DOI:** 10.3390/md15070198

**Published:** 2017-06-25

**Authors:** Pedro L. Flores, Emma Rodríguez, Estrella Zapata, Roxana Carbó, José María Farías, Martín Martínez

**Affiliations:** 1 Departamento de Instrumentación Electromecánica, Instituto Nacional de Cardiología “Ignacio Chávez”, Juan Badiano # 1, Col. Sección XVI, México City 14080, Mexico; pelfoch07@yahoo.com.mx; 2 Laboratorio de Biología Celular, Departamento de Fisiología, Instituto Nacional de Cardiología “Ignacio Chávez”, Juan Badiano # 1, Col. Sección XVI, México City 14080, Mexico; emarod2@yahoo.com.mx (E.R.); estrella_zg@yahoo.com (E.Z.); 3 Departamento de Biomedicina Cardiovascular, Instituto Nacional de Cardiología “Ignacio Chávez”, Juan Badiano # 1, Col. Sección XVI, México City 14080, Mexico; roxcarbo@gmail.com; 4 Departamento de Fisiología, Facultad de Medicina, Universidad Nacional Autónoma de México, México City 04510, Mexico; chema_farias@hotmail.com; 5 Departamento de Fisiología, Instituto Nacional de Cardiología “Ignacio Chávez”, Juan Badiano # 1, Col. Sección XVI, México City 14080, Mexico

**Keywords:** *Xenopus laevis* oocytes, maitotoxin, ciguatera fish poisoning, TRPC channels

## Abstract

Maitotoxin (MTX) is the most potent marine toxin known to date. It is responsible for a particular human intoxication syndrome called *ciguatera fish poisoning* (CFP). Several reports indicate that MTX is an activator of non-selective cation channels (NSCC) in different cell types. The molecular identity of these channels is still an unresolved topic, and it has been proposed that the transient receptor potential (TRP) channels are involved in this effect. In *Xenopus laevis* oocytes, MTX at picomolar (pM) concentrations induces the activation of NSCC with functional and pharmacological properties that resemble the activity of TRP channels. The purpose of this study was to characterize the molecular identity of the TRP channel involved in the MTX response, using the small interference RNA (siRNA) approach and the two-electrode voltage-clamp technique (TEVC). The injection of a specifically designed siRNA to silence the transient receptor potential canonical type 1 (TRPC1) protein expression abolished the MTX response. MTX had no effect on oocytes, even at doses 20-fold higher compared to cells without injection. Total mRNA and protein levels of TRPC1 were notably diminished. The TRPC4 siRNA did not change the MTX effect, even though it was important to note that the protein level was reduced by the silencing of TRPC4. Our results suggest that MTX could be a selective activator of TRPC1 channels in *X. laevis* oocytes and a useful pharmacological tool for further studies on these TRP channels.

## 1. Introduction

Maitotoxin (MTX) is produced by the microscopic algae named *Gambierdiscus toxicus*, and is the most potent marine toxin known to date [1,2]. The lethal dose for mice is less than 0.2 µg/kg IP. MTX is at least five-fold more toxic than tetrodotoxin, which is another very well-known marine toxin [3,4,5]. MTX is one of the toxins that cause a human intoxication syndrome called *ciguatera fish poisoning* (CFP), which is the most common cause of seafood-toxin illness in the world. CFP produces gastrointestinal, neurologic, and cardiovascular symptoms which last from days to weeks or even months [6]. Climate change has expanded the regions near the equator where *G. toxicus* lives [7,8,9,10]. Furthermore, due to the increase in trade, worldwide seafood consumption, and international tourism, CFP can happen at any location [6,11,12]. Several other ciguatoxins and their mechanisms of action have been identified, but the mechanism of MTX remains unknown [13]. Multiple reports have indicated that MTX is an activator of non-selective cation channels (NSCC) in different cell types [14,15,16,17,18], and it has been suggested that the channels belonging to the transient receptor potential (TRP) superfamily are involved in its effect. The TRP channel family contains 28 members and is subdivided into six subfamilies: TRPA, TRPC, TRPML, TRPM, TRPN, TRPV, and TRPP, all of which permit the permeation of cations [19]. The TRP channels have many physiological roles: they participate as sensors of temperature, pH, and mechanical stress, as well as in some human diseases [20,21]. The canonical transient receptor potential channels (TRPCs) are the first encoded TRP gene family discovered in mammals, and are the most dominant non-voltage-gated cation channels in various cells [22]. Two TRP channels—TRPC1 and TRPC4—have been found in *X. laevis* oocytes, where TRPC1 is a mechanosensitive channel in the frog oocyte membrane [23,24].

The TRPC1 of *X. laevis* oocytes is highly homologous to the human isoform of TRPC1 (hTRPC1) [23,25,26], and MTX is an initiator of hTRPC1 activation, which has a heterogenous expression in the H4-IIE rat liver cell line [17]. In *X. laevis* oocytes, the NSCC activation by MTX has been characterized [15,18], with these channels having functional and pharmacological properties that resemble TRP channels [17,18]. The aim of this study is to search the identity of the putative TRP channel involved in the MTX response using TRPC1 and TRPC4 small interference RNA (siRNA) in addition to the two-electrode voltage-clamp technique (TEVC).

## 2. Results

### 2.1. Currents Induced by MTX in *X. laevis* Oocytes

**Figure 1:**
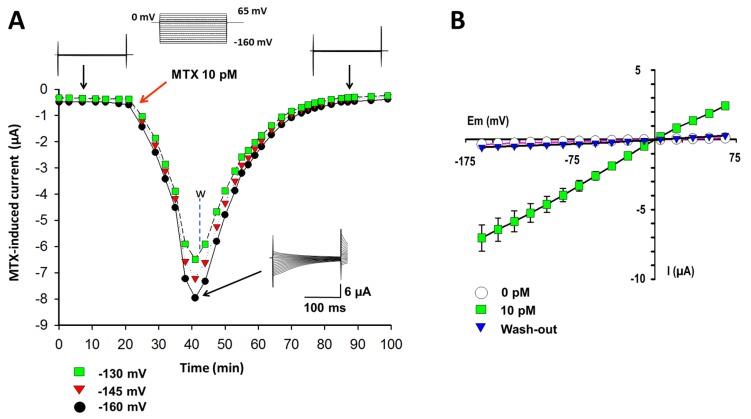
Currents induced by maitotoxin (MTX) in control oocytes. (**A**) Temporal course of currents induced by 10 pM MTX obtained by the two-electrode voltage-clamp technique (TEVC) and with a hyperpolarizing protocol of pulses at three membrane voltages (−160, −145, and −130 mV). MTX was added at the point indicated by the red arrow. The MTX-induced currents showed a maximal effect in about 15 min. MTX was washed out by perfusion with Ringer solution, and complete recovery was reached after 45 min. Representative recordings traced before adding MTX, at the maximal current level, and at the end of a continuous washing are shown as inserts. (**B**) Current–voltage (I–V) relationships for the peak of currents obtained for all three conditions (control, MTX, and recovery) are shown (*n* = 3). The symbols represent the means, and the bars represent the standard error of the mean (s.e.m.).

The first goal was to reproduce the MTX effects in oocytes following similar conditions to those previously reported [15,18]. Using the TEVC technique and a hyperpolarizing protocol of pulses, basal currents were recorded in the Ringer solution. When 10 pM MTX was added to the bath, a time-dependent conductance was induced, reaching its maximal level in approximately 15 min (Figure 1A). The maximal current at this concentration and its kinetics corresponds to previously reported values [15,18]. To prove the integrity of the membrane, the MTX was washed with a Ringer solution, and the effect was completely reversible after 45 min.

The current-voltage (I–V) relationships obtained at the basal condition, at the maximal current at 10 pM of MTX, and a recovery period in three independent experiments are shown in Figure 1B. MTX-induced currents are several times greater than the basal level. The I–V curves present a linear ohmic behavior and a reversal potential near to zero, which is characteristic of NSCC and TRP channels [20]. The currents were permeable to monovalent cations (Na+ and K+) with a higher selectivity for K+ (data not shown) as previously proven [15,18].

### 2.2. Identification of the MTX Current

To identify the molecular nature of the MTX-induced current in *X. laevis* oocytes, TRPC1 and TRPC4 siRNAs designed for specifically silencing each one of these channels were injected into oocytes before the MTX effect was tested.

**Figure 2:**
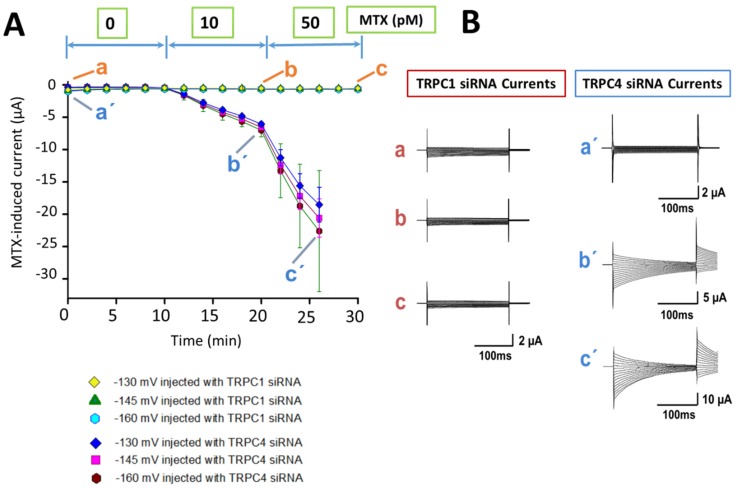
Effects of MTX in oocytes injected with transient receptor potential channels 1 and 4 (TRPC1 and TRPC4) siRNAs. (**A**) Temporal course of the MTX effects in oocytes injected with TRPC1 and TRPC4 siRNAs at day three after injection. The symbols represent the mean (±SD) of the maximal current obtained at three membrane voltages (−160, −145, and −130 mV) in three independent experiments. In oocytes injected with TRPC1 siRNA, MTX did not induce evident currents at 10 and 50 pM. The mean of currents from oocytes injected with TRPC4 siRNA was similar to non-injected oocytes. (**B**) Representative recording traces for each condition. The current traces from oocytes injected with TRPC1 siRNA were obtained at points a, b, c (in red), as well as the current traces from oocytes injected with TRPC4 siRNA in points a’, b’, and c’ (in bue) are shown.

Figure 2A shows the temporal course of the MTX response applied to the oocytes injected with TRPC1 siRNA and TRPC4 siRNA. MTX did not induce any current at 10 and 50 pM in oocytes injected with TRPC1 siRNA. Higher toxin concentrations (10–200 pM) were tested, and no currents were induced. Only up to a toxin concentration of 500 pM, the currents generated showed amplitudes in the range of the leak current (data not shown). In oocytes injected with TRPC4 siRNA, the temporal course of the MTX effect showed a behavior similar to the control. Essentially, MTX-induced currents at 10 and 50 pM were developed in the same range of amplitudes as in the non-injected oocytes [18]. Figure 2B shows representative current traces for each condition in Figure 2A. The basal levels of current from oocytes injected with TRPC1 siRNA did not change with the application of MTX in the whole range of doses. The MTX-induced currents obtained from TRPC4 siRNA-injected oocytes showed amplitude and kinetics similar to recording traces in non-injected oocytes.

### 2.3. Expression of TRPC mRNA and Protein

**Figure 3:**
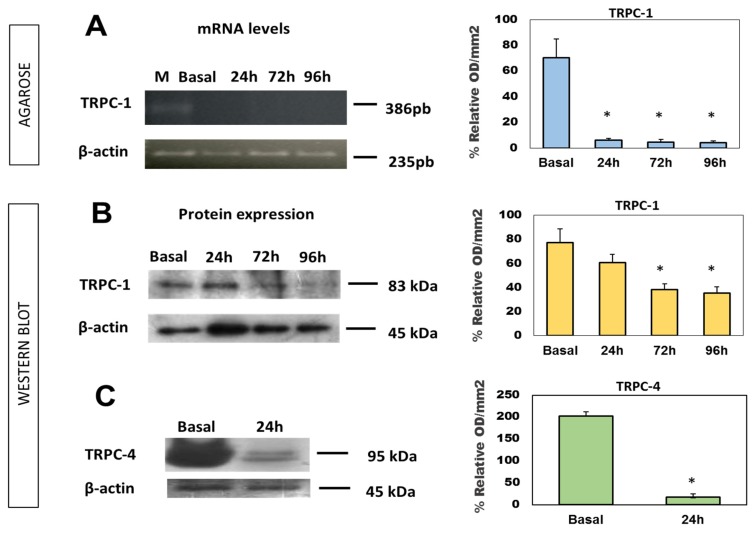
TRPC1 and TRPC4 expression in injected oocytes. (**A**) TRPC1 mRNA levels at different times of incubation after TRPC1 siRNA injection; (**B**) and (**C**) show the protein expression levels for TRPC1 and TRPC4, respectively. Values are the results of the means ± SD, *n* = 3; each experiment was formed with at least 15 oocytes. A one-way analysis of variance (ANOVA) was performed, followed by a Mann–Whitney test.

The results from TRPC1 mRNA expression as well as TRPC1 and TRPC4 protein levels in injected oocytes are shown in Figure 3. Figure 3A represents the TRPC1 mRNA expression in an agarose gel and its chart representation at 24, 72, and 96 h of incubation after the TRPC1 siRNA injection. The TRPC1 mRNA level was reduced by 91.4 ± 1.5, 93.4 ± 2.2, and 93.9 ± 1.3% at 24, 72, and 96 h after injection, respectively. Figure 3B,C shows the reduction in protein levels of TRPCs in an acrylamide gel and their corresponding charts. The decrease in the expression levels of the TRCP1 protein is less evident than those of the mRNA expression levels (chart in Figure 3A). These were 21.5 ± 6.8, 50.6 ± 4.5, and 54.3 ± 5.2% at 24, 72, and 96 h of treatment respectively, which also showed a tendency to decrease over time (Figure 3B). Finally, Figure 3C represents the protein levels from TRPC4 siRNA-injected oocytes, which were reduced by up to 91.49 ± 1.7% after 24 h.

### 2.4. Intracellular Calcium Participation in the MTX Effects

**Figure 4:**
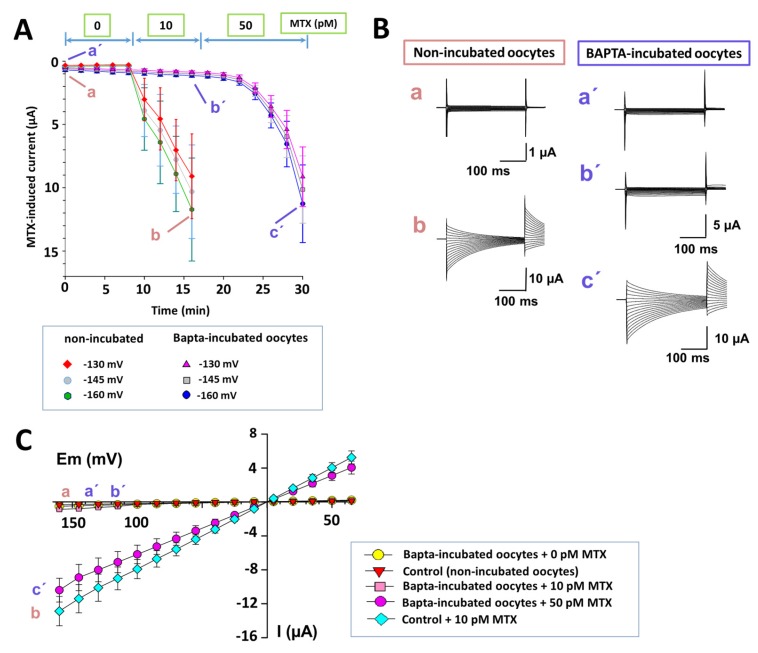
MTX effect on N,N′-[1,2-ethanediylbis(oxy-2,1-phenylene)]bis[N-[2-[(acetyloxy)methoxy]-2-oxoethyl]]-, bis[(acetyloxy)methyl] ester (BAPTA-AM (BAPTA-AM)-incubated oocytes. (**A**) Temporal course of MTX effect (10 and 50 pM) on pre-incubated oocytes with 200 nM BAPTA-AM and on non-incubated oocytes. The maximal current level (mean ± SD; *n* = 4) at three membrane voltages are shown (−160, −145, and −130 mV) for incubated and non-incubated oocytes. (**B**) Representative current traces of the MTX effect extracted from the temporal course experiments are indicated for each condition. (**C**) The current–voltage relationships for the maximal current (mean ± s.e.m.) for incubated and non-incubated cells are shown (*n* = 4).

The intracellular calcium participation in MTX effects has been previously demonstrated [14,16,27,28,29,30]. Therefore, the oocytes were incubated with 200 nM of glycine, N,N′-[1,2-ethanediylbis(oxy-2,1-phenylene)]bis[N-[2-[(acetyloxy)methoxy]-2-oxoethyl]]-, bis[(acetyloxy)methyl] ester (BAPTA-AM: a highly selective cell-permeant calcium chelator) for 12 h. After washing the cells with a Ringer solution, MTX effects were tested. Figure 4A shows the temporal course of MTX effects (10 and 50 pM) in four BAPTA-incubated oocytes compared with MTX effects (10 pM) in four non-incubated oocytes. The MTX effect in BAPTA-incubated cells was reduced compared with non-incubated cells. Essentially, MTX effects produced at 50 pM in BAPTA-incubated oocytes were similar to the effects produced by MTX at 10 pM in non-incubated oocytes. Furthermore, a delay in the MTX effect was produced in BAPTA-incubated oocytes. Figure 4B shows representative current recordings from each condition. Figure 4C shows the I–V relationships for BAPTA-incubated and non-incubated oocytes for each MTX concentration.

## 3. Discussion

The results indicate that TRPC1 channels could be the molecular counterpart of MTX-activated NSCC in *X. laevis* oocytes. The range of MTX doses (pM) used suggests a highly specific interaction between the toxin and TRPC1 channels. The interaction at the pM range of MTX with hTRPC1 has previously been observed, and it was proposed that this channel could be a target for MTX [17]. Considering that hTRPC1 is highly homologous (84% identical and approximately 90% similar in amino acids) to *X. laevis* TRPC1 [23,25,26], it is possible to speculate a selective effect on TRPC1 in *X. laevis* oocytes. The present results demonstrate that the TRPC1 siRNA used can ablate TRPC1 protein expression. Therefore, it is very likely that the endogenous NSCC activated by MTX in oocytes could be the TRPC1 channel. These findings have a correlation with the functional role of this channel in *X. laevis* oocytes, which forms a stretch-activated cation channel [24].

The mRNA and protein levels of TRPC1 were notably diminished in oocytes injected with TRPC1 siRNA. TRPC1 mRNA was reduced by more than 90% after 24 h. The reduction in protein level was lower than the reduction in mRNA levels. However, the levels were similar to those reported in liver cells using siRNA to silence TRPC1 [31]. Additionally, the TRPC1 protein level was inhibited progressively through time after injection with TRPC1 siRNA, suggesting an effect that was dependent on the turnover rate of the protein channel expression. On the other hand, the injection of TRPC4 siRNA did not affect the MTX response. Essentially, these currents were similar to those found in control conditions, even with a practically null TRPC4 protein expression. In this regard, the TRPC4 siRNA assay could be considered as a positive control of the injection protocol.

The TPRC1 localization in the oocyte membrane is equal to that of all TRPCs [32]. In the present results, the MTX effect is not immediate. The effect occurs in a range of minutes as in other cell types [13,27,28,30], which could suggest that another molecule(s) might be participating in the response, possibly a membrane receptor and its associated intracellular pathway. In this sense, the TRPC channels can be regulated by G-protein-coupled receptors (GPCRs) including lipid signals [33]. As MTX applied to *X. laevis* oocytes triggers the breakdown of phosphatidylinositol 4,5-bisphosphate, this increases the intracellular Ca^2+^ concentration and mimics fertilization [34].

The results from oocytes incubated with BAPTA-AM demonstrate that the currents induced by MTX were at a lower level and had a slower course of the effect compared to controls. This behavior suggests that intracellular calcium has a role in the MTX response. In addition, the I–V relationships of the MTX-induced currents from BAPTA-AM-incubated oocytes continued to be linear ohmic and the reversal potential did not change, suggesting that no other cation is permeating. The entry of Ca^2+^ through the TRPC1 channel is limited because it has been described that MTX-induced NSCC has a small Ca^2+^ permeability [15,17,18], being mainly permeable to monovalent cations (Na+, K+, and Cs+) [15,18,24]. The characterization of this part of the action mechanism is exciting, but further studies are required.

Several reports demonstrate that TRPC channels tend to form heterodimers, such as TPRC1 with TRPC5 and TPRC1 with TRPC6, in addition to other combinations [31,35,36,37,38,39]. However, it is not clear if TRPC1 can form functional homodimers [38]. The present results demonstrated that TRPC1 and TRPC4 proteins are expressed in *X. laevis* oocytes. However, the possibility of TRPC1 forming heterodimers with TRPC4 is limited since in the TRPC4 siRNA-injected oocytes (which shows an almost null presence of TRPC4 protein level), there is no difference in the effects of MTX when comparing it to control oocytes. Thus, the current is developed only through a homomeric TRPC channel.

Finally, considering that TRPC1 is the most important channel involved in MTX-induced currents in *X. laevis* oocytes and the low pM concentration in which MTX is capable of producing the effect, this toxin could be considered as a selective pharmacological tool for TRPC1 activation studies.

## 4. Material and Methods

### 4.1. Animals and Ethical Statement

African clawed frogs (*Xenopus laevis*) of 12 months old (125–150 g, females) were used (NASCO; Biology Div., Fort Atkinson, WI, USA). The experimental animals were kept in a fishbowl under a controlled temperature (23 ± 2 °C) and air regulation. These animals were fed with commercial frog food (NASCO; Biology Div., Fort Atkinson, WI, USA), considering the animal’s circadian cycle. All procedures were performed following the ethical standards of the responsible committee on animal experimentation. Animals were lawfully acquired, and the manipulation of the research was carried out by “The Code of Ethics of the World Medical Association” (Declaration of Helsinki): EC Directive 86/609/EEC for animal experiments [40]. The protocol was reviewed and approved by the Ethics Committee of the Instituto Nacional de Cardiología “Ignacio Chávez.” Our experimental work followed the guidelines of the Norma Official Mexicana guide for the use and care of laboratory animals (NOM-062-ZOO-1999) and the disposal of organic residues (NOM-087-ECOL-1995). The authors have read the International Association of Veterinary Editors’ Consensus Author Guidelines on Animal Ethics and Welfare, declaring that all the animals used in this study were treated according to the statement mentioned above. All reagents were of analytical quality and obtained from Sigma-Aldrich (St. Louis, MO, USA), aside from others that were specifically indicated.

### 4.2. Oocytes

Defolliculated oocytes were collected from each female frog according to a previous study [41]. Briefly, frogs were anesthetized by immersion in a 0.17% tricaine aqueous solution for 30 min. Small pieces of the ovarian lobe were obtained and gently shaken at room temperature (19–25 °C) for 90 min in a Ca2+-free frog Ringer (ND96) solution. This solution contained 96 mmol/L of NaCl, 2.5 mmol/L of KCl, 1 mmol/L of MgCl_2_, 5 mmol/L of HEPES (4-(2-hydroxyethyl)-1-piperazineethanesulfonic acid) at a pH of 7.4, which was supplemented with 2 mg/mL of collagenase type IA. Three hundred oocytes were collected from each frog, with only healthy-looking oocytes of V–VI stages being used. Oocytes were incubated at 18 °C for 1–5 days in the ND96 solution supplemented with 2.5 mM of pyruvic acid, 20 U/mL of gentamycin, and 20 mg/mL of streptomycin. The incubation solution was changed daily. Frogs recovered from the surgical procedure were returned to the fishbowl. Some oocytes were incubated with 200 nM of BAPTA-AM (Glycine, *N*,*N*′-[1,2-ethanediylbis(oxy-2,1-phenylene)]bis[*N*-[2-[(acetyloxy)methoxy]-2-oxoethyl]]-, bis[(acetyloxy)methyl] ester) before being dissolved in the Ringer solution at 18 °C for 12 h to investigate the participation of intracellular calcium. After the incubation time, the cells were washed with the Ringer solution and were immediately recorded.

### 4.3. Electrophysiology

Electrophysiological recordings were made in a 1 mL chamber. Each oocyte was impaled in the Ringer solution containing 117 mmol/L of NaCl, 2.5 mmol/L of KCl, 1.8 mmol/L of CaCl_2_, 5 mmol/L of HEPES at a pH of 7.4 and at room temperature (19–25 °C). Electrodes showed resistances of 0.5–1 MΩ (3 M of KCl). Currents were recorded using the TEVC technique, with a −160 to 65 mV protocol implemented in steps of 15 mV (Hp = 0 mV) using a Geneclamp 500B amplifier (Axon Instruments Inc., Foster City, CA, USA). The signal was digitized at a rate of 10 kHz with an AD/DA converter (Digidata 1322A, Axon Instruments Inc., Foster City, CA, USA) and filtered at a rate of 2–5 kHz. Data was stored and was analyzed offline on a personal computer using pCLAMP v. 8.0 (Axon Instruments Inc., Foster City, CA, USA), Microsoft Excel, v. 2010 and SigmaPlot, v. 9.01 (Jandel Scientific, San Jose, CA, USA). The MTX (Wako Chemicals, Osaka, Japan) was added to the recording chamber to get the final concentration (10–500 pM), which was previously dissolved with bovine serum albumin to obtain a homogenized solution. The external solution was changed by a perfusion system.

### 4.4. Small Interference RNA (siRNA) Assay

siRNA duplexes for *X. laevis* TRPC1 (GenBank: NM_001090350) and mouse TRPC4 (GenBank: NM_016984) were designed by Ambion Inc. (Life Technologies, Carisbad, CA, USA) per the instructions of a previous study [24]. Individual TRPC1 siRNA duplexes were oligo1 (sense sequence 5′–3′) with GCA, GCA, AAG, CAA, CAA, CACUtt; and oligo2 (antisense sequence 5′–3′) with AGU, GUU, GUU, GCU, UUG, CUGCtc. For TRPC4, the oligos were (sense sequence 5′–3′) CCU UGA AGA UUG UCG CGUUtt and (antisense sequence 5′–3′) AAC GCG ACA AUC UUC AAGGag. At time 0, oocytes were injected with siRNA oligonucleotides against TRPC1 and TRPC4 (50 ng per cell). Recordings of MTX-induced currents were made at 24, 72, and 96 h after injection.

### 4.5. RT-PCR

Total RNA was extracted from pools of 15 oocytes injected with TRPC1 siRNA and incubated during 24, 72, and 96 h. The oocytes pools were lysed with 1 mL of Trizol (Molecular Research Center Inc., Cincinnati, OH, USA) to obtain total RNA, according to manufacturer instructions. Following this, it was resuspended in diethyl pyrocarbonate-treated water, and its concentration was determined in NanoDrop 1000 (Thermo Scientific, Waltham, MA, USA) at 260 nm. A total of 1 µg of total RNA was used to make the RT-PCR reaction. The first complementary DNA chain (cDNA) synthesis was achieved by using Thermo Scientific Kit (K1622, Thermo Scientific, Waltham, MA, USA). The cDNA mixture was incubated at 37 °C for 50 min and then at 70 °C for 15 min. The resulting cDNA was amplified by PCR with a recombinant Taq polymerase (Invitrogen). The sequences for TRPC1 were 5′–CCG GGC CAA GCT CTG GTT GA–3′ (upstream) and 5′–GCG ATG CAC AAG GCA GCA CA–3′ (downstream), with an amplified product of 235 bp obtained. The conditions for PCR were 95 °C for 45 s (denaturing), 63 °C for 1 min (annealing) and 72 °C for 1 min (extension) in the Veriti Thermal Cycler (Applied Biosystems, Foster City, CA, USA). The sequences for β-actin (as an internal control) were 5′–GTG ACC CGC CCG CAT AGA AAG–3′ (upstream) and 5′–CAG GGG TGC TTC TGT GAG CAG CAG C–3′ (downstream), with an amplified product of 391 bp obtained. After 30 such cycles, PCR products were detectable by 2% agarose-ethidium bromide gel electrophoresis, while the bands obtained were analyzed by a GS-800 calibrated densitometer (Bio-Rad, Irvine, CA, USA).

### 4.6. Western Blotting

Fifteen oocytes from each experimental condition were recovered and homogenized with 300 µL of lysis buffer (20 mM Tris-HCl, 137mM NaCl mM, 2 EDTA, 2% SDS) at a pH of 7.5 and using protease inhibitors mix (Sigma-Aldrich, St. Louis, MO, USA). After homogenization, the samples were centrifuged at 14,000 rpm for 15 min. The protein content of supernatants was quantified using the Bradford method [42]. Vertical slab by 10% sodium dodecyl sulfate-polyacrylamide gel electrophoresis (SDS-PAGE) using Tris-glycine was performed, and 50 µg/mL of the protein sample was employed in each lane. Gel proteins were transferred onto PVDF membranes at 130 V for 70 min. Membranes were incubated in 5% nonfat dry milk at 4 °C overnight before being subsequently rinsed three times in PBS-Tween (0.1%) at a pH of 7.4 for 15 min each. Following this, the membranes were incubated with three primary antibodies at room temperature for 2 h. This study used a TPRC1 anti-goat polyclonal antibody (1:200) (Santa Cruz Biotechnology, Dallas, TX, USA), a TPRC4 anti-rabbit polyclonal antibody (1:200) (ENZO Life Technology, Farmingdale, NY, USA), and a β-actin anti-rabbit polyclonal antibody (1:1000) (Santa Cruz Biotechnology, Dallas, TX, USA) as load control. Membranes were washed again with PBS-TWEEN and incubated with a secondary antibody (anti-goat or anti-rabbit IgG-HRP, 1:1000) at 37 °C for 1.5 h. The bound antibodies were visualized with Immobilon Western Chemiluminescent HRP Substrate (Merck Millipore, Billerica, MA, USA), using classical photo revealing. Signal density was obtained by scanning the blots on a Bio-Rad imaging system (GS-800 Calibrated Densitometer, Bio-Rad laboratories, Hercules, CA, USA). Normalized density was calculated by dividing the rough density values of a sample band over the loading control band (β-actin). A single band for each protein with a molecular weight of 83 kD for TRPC1, 95 kD for TRPC4 and 45 kD for actin was observed.

### 4.7. Statistical Analyses

Electrophysiological experimental data are reported as the means ± standard error (s.e.m) or ±standard deviation (SD). Protein expression values from the Western blot are the results of the means ± standard deviation (SD), *n* = 3. Each experiment was formed with at least 15 oocytes (*p* < 0.05). A one-way analysis of variance (ANOVA) was performed, followed by a Mann–Whitney test.

Differences in mRNA expression values by RT-PCR are the results of the means ± standard deviation (SD), with each experiment formed with at least 15 oocytes *n* = 3; (*p* < 0.05). A one-way ANOVA was performed; followed by a Mann–Whitney test. The Graph Pad Prism software (v 5.00, Graph Pad, San Diego, CA, USA) was used to perform the analyses.
